# Hybrid Bionic Nerve Interface for Application in Bionic Limbs

**DOI:** 10.1002/advs.202303728

**Published:** 2023-10-15

**Authors:** Youngjun Cho, Hyung Hwa Jeong, Heejae Shin, Changsik John Pak, Jeongmok Cho, Yongwoo Kim, Donggeon Kim, Taehyeon Kim, Hoijun Kim, Sohee Kim, Soonchul Kwon, Joon Pio Hong, Hyunsuk Peter Suh, Sanghoon Lee

**Affiliations:** ^1^ Department of Robotics and Mechatronics Engineering Daegu Gyeongbuk Institute of Science and Technology (DGIST) Daegu 42899 South Korea; ^2^ Department of Plastic and Reconstructive Surgery Asan Medical Center, University of Ulsan College of Medicine 05505 Seoul South Korea; ^3^ Graduate School of Smart Convergence Kwangwoon University Seoul 01897 South Korea

**Keywords:** neural interface, neuroprosthetic, regenerative peripheral nerve interface, robotic leg, shape memory polymer

## Abstract

Intuitive and perceptual neuroprosthetic systems require a high degree of neural control and a variety of sensory feedback, but reliable neural interfaces for long‐term use that maintain their functionality are limited. Here, a novel hybrid bionic interface is presented, fabricated by integrating a biological interface (regenerative peripheral nerve interface (RPNI)) and a peripheral neural interface to enhance the neural interface performance between a nerve and bionic limbs. This interface utilizes a shape memory polymer buckle that can be easily implanted on a severed nerve and make contact with both the nerve and the muscle graft after RPNI formation. It is demonstrated that this interface can simultaneously record different signal information via the RPNI and the nerve, as well as stimulate them separately, inducing different responses. Furthermore, it is shown that this interface can record naturally evoked signals from a walking rabbit and use them to control a robotic leg. The long‐term functionality and biocompatibility of this interface in rabbits are evaluated for up to 29 weeks, confirming its promising potential for enhancing prosthetic control.

## Graphical abstract

A hybrid bionic interface that integrates a biological interface and a neural interface is developed, demonstrating simultaneous nerve and muscle graft contact. Chronic implantation in a rabbit model validates stable recording and stimulation. Long‐term implantation (29 weeks) is validated. Multiple signal acquisition possibilities and potential neuroprosthetic applications are highlighted, with an emphasis on future advancements in bionic interface technology.

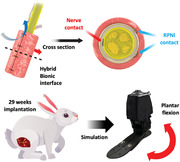

## 1 Introduction

In the US and Europe, amputation is performed annually on 242,230 and 87,088 individuals, respectively.^[^ ^1^ ^]^ Neuroprosthetics, which are robotic limbs used to replace lost body parts and their functions, have been developed to recover patients’ quality of life. However, to completely replace amputated limbs with bionic limbs that offer motor function and sensory feedback, a bidirectional connection to the nervous system is necessary. A strategy of connecting to the peripheral nervous system via neural interfaces has shown significant results in achieving this goal. The motor function of bionic arms has been restored to fine‐grained movements, such as individual finger movements, through connecting with nerves via neural interfaces.^[^ ^2^ ^]^ Sensory feedback is also critical for neuroprosthetics. Various methods, such as haptic feedback,^[^ ^3^ ^]^ somatosensory feedback,^[^ ^4^ ^]^ and neural sensory feedback,^[^ ^5^, ^6^, ^7^, ^8^, ^9^ ^]^ have been used to restore sensory feedback, which helps to restore lost sensation in residual limbs and enable more natural and accurate prosthetic control through sensory feedback.^[^ ^10^, ^11^ ^]^ Sensory feedback also improves mobility, prevents falls, and promotes agility in lower‐limb neuroprosthetics.^[^ ^12^ ^]^ Sensory feedback through the restoration of sensation can also be a therapeutic solution for phantom pain caused by the lack of physiological feedback from residual limbs after amputation.^[^ ^13^, ^14^ ^]^

However, the main challenge of directly interacting with the peripheral nervous system is the stable and long‐term implantation of neural interfaces that maintain their functionality for continuous communication with neuroprosthetics. The materials of peripheral nerve interfaces should be biocompatible and minimize mechanical mismatch with neural tissues to minimize the immune response that can occur during chronic implantation.^[^ ^15^ ^]^ Furthermore, various factors, such as the implantation method, electrode position, and number of electrodes, should be carefully considered in motor control and sensory feedback strategies. Despite the use of soft materials, such as silicone materials^[^ ^16^, ^17^ ^]^ and flexible thin‐film materials,^[^ ^2^, ^18^, ^19^, ^20^, ^21^ ^]^ at present, there are no neural interfaces that meet these requirements.

To overcome the limitations of engineered interfaces, biological interfaces have been suggested to meet the requirements of long‐term reliability. Regenerative peripheral nerve interfaces (RPNIs) can be implanted via a surgical technique to provide a muscle graft at the nerve endings and have shown positive results in reducing phantom pain and neuroma.^[^ ^22^, ^23^ ^]^ RPNIs have enabled the stable and independent acquisition of neural signals as a peripheral nerve interface and have shown positive results in neural prosthetic control and the restoration of proprioception and cutaneous sensation.^[^ ^2^, ^24^ ^]^ Recently, the concept of the cutaneous mechanoneural interface (CMI) was reported, which merges advanced reconstructive surgery techniques with a sensorized prosthesis to achieve the restoration of sensory cutaneous feedback.^[^ ^25^ ^]^ However, accessing these biological interfaces still requires wires, such as stainless wires, for the acquisition of biological signals or for stimulation.^[^ ^26^ ^]^ This can cause issues when the number of interfaces increases at distal parts for selectivity, as this would also require the number of wires to increase. Furthermore, at proximal parts, CMI techniques show limited selectivity since they indirectly connect with nerves. Therefore, a strategy for maximizing neural interface performance using their merits is required to achieve the ultimate goal.

**Figure 1:**
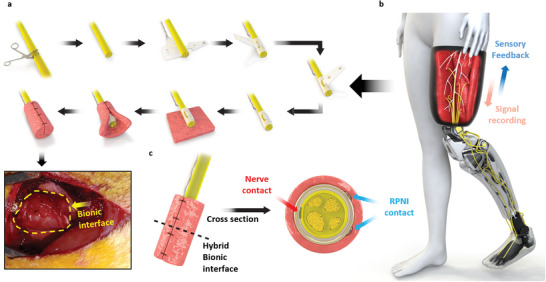
Schematic of the hybrid bionic interface. a) Schematic diagram of the process of bionic interface formation. First, the nerve is severed, and the head of the buckle is inserted into the buckle hole and tightened. Next, the head of the buckle is oppositely folded 180 degrees and wrapped around the nerve. Muscle tissue is then segmented, wrapped around the implanted neural interface and nerve, and treated to form the RPNI. See also a photo of a bionic interface from the rabbit model. b) Conceptual illustration of the formation of bionic interfaces providing sensory feedback and motor control for neural prosthetic control in amputee patients. c) Cross‐sectional view of the contact position of the buckle interface.

Here, we demonstrate a hybrid bionic interface that combines a biological neural interface (RPNI) and a peripheral neural interface to enhance the diversity of recording and stimulation capabilities by simultaneous nerve and muscle graft contact with one interface. As a proof‐of‐concept, we develop a buckle‐shaped neural interface using a shape memory polymer (SMP) mimicking a buckle strap. This design allows easy and quick implantation on a severed nerve. Furthermore, it provides contact to the nerve and the muscle graft after RPNI formation (**Figure** **1**a). This bionic interface ideally not only enables the simultaneous recording of different types of neural information on the user's intention via the RPNI and the nerve but can also separately stimulate the RPNI and the nerve, diversifying sensory feedback (Figure 1b,c). To prove this concept, electrically evoked signals (induced efferent signals) by a cuff electrode in a rabbit sciatic branch nerve are simultaneously recorded by the bionic interface RPNI (BI‐R) area and bionic interface nerve (BI‐N) area and are analyzed to evaluate the correlation. During long‐term experiments up to 20 weeks, the functionality of stimulation by BI‐R and recording by BI‐N and vice versa are demonstrated to investigate the different roles of each electrode and the reliability of the bionic interface formation. Furthermore, we conduct a recording experiment of naturally evoked efferent signals during the walking of rabbits, the data of which is analyzed and applied to operate a robotic leg. Finally, chronic implantation in rabbits for 29 weeks is performed to demonstrate the biocompatibility of the implanted neural interface and the formation of the RPNI, and a histological test is conducted.

## 2 Result and Discussion

### 2.1 Buckle‐Shaped Neural Interface

**Figure 2:**
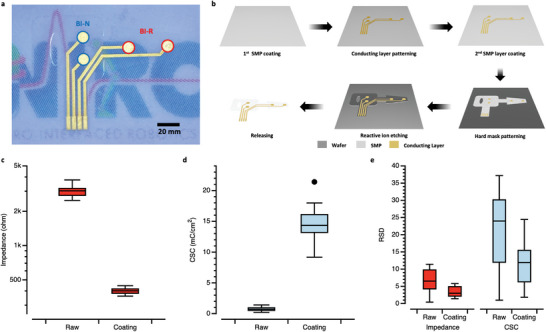
Fabrication and characterization of the bionic interface a) Photo of the fabricated SMP buckle interface with a total of 4 channels in one neural interface. b) Fabrication process of the buckle interface. c) Impedance changes of all channels (*n* = 37) before and after IrO2 coating. (d) CSC changes of all channels (*n* = 37) before and after coating. e) Changes in the RSD of the impedance and the CSC before and after coating. The RSD of one interface was calculated to determine how uniform the performance level was across all channels for a consistent recording configuration. The RSDs of a total of 11 electrodes were calculated.

We designed a buckle‐shaped neural interface for achieving simultaneous contact with a severed nerve and a muscle graft. The SMP interface was fabricated by a microelectromechanical system (MEMS) fabrication process that also included an advantageous reliable micropatterning process for achieving a thickness of several tens of micrometers.^[^ ^27^ ^]^ At room temperature, the fabrication material was flexible (1.5 GPa), and it could soften to 400 MPa at body temperature. It was also biocompatible,^[^ ^28^ ^]^ making it suitable for fabricating peripheral nerve interfaces.^[^ ^29^ ^]^ **Figure** **2**a shows the fabricated SMP interface, which had two electrodes for making parallel contact with the nerve and two electrodes for making contact with the muscle graft. The fabricated SMP electrode was transparent, allowing visibility for surgery and using other types of measurement, such as imaging. The detailed fabrication process is described in the Methods section (Figure 2b). Furthermore, for reliable chronic implantation, a wire was packed in a helical structure to withstand a maximum strain of 110% without tearing and impedance change. The detailed packaging process is shown in Figure S1 (Supporting Information) and the strain test in Figure S2 (Supporting Information).

To enhance the electrochemical performance and reliability of the interface, iridium oxide was additionally coated on an Au electrode. The size of the coated electrode was 0.5026 mm^2^. The performance of the buckle interface before and after IrO_2_ coating was investigated through impedance and cyclic voltammetry (CV). A total of 11 buckle interfaces were investigated, and the results showed that the impedance decreased by 7.206 times from 3.017 to 0.419 kΩ at 1 kHz (Figure 2c), and the charge storage capacity (CSC) increased by 19.311 times from 0.746 to 14.404 mA cm^−2^ (Figure 2d; Figure S3, Supporting Information). These performance changes can be expected to increase the quality of signal recording and increase the efficiency of stimulation by increasing the charge injection capacity.^[^ ^30^, ^31^ ^]^

For the recording of naturally evoked or electrically evoked neural signals, one crucial factor is improving the signal‐to‐noise ratio (SNR) through noise reduction. Differential bipolar or tripolar recording configurations are typically proposed for noise reduction, especially for extra‐neural recording.^[^ ^32^, ^33^ ^]^ In these configurations, accurate neural signal acquisition requires using working and reference electrodes with the same performance. In this regard, precise improvement in the electrochemical performance of each electrode in a multichannel neural interface is needed. Accordingly, the relative standard deviation (RSD) was investigated for all channels (*n* = 4) of one interface, and the average RSD of all interfaces (*n* = 11) was calculated to confirm the precise change of the interface before and after coating. As a result, the average RSD of the CSC for each channel (*n* = 4) of the interfaces decreased from 20.89% to 11.62% after coating, and the impedance decreased from 7.57% to 6.84% after coating (Figure 2e). This result reveals that the IrO_2_ coating can improve and standardize the neural interface performance by precisely improving the performance of each channel.

### 2.2 Electrical Evoked Signal Recording

#### 2.2.1 Investigation of Optimal Stimulation Parameters for Neural Signal Acquisition at Normal Rabbits

Electrophysiological evaluation was conducted at normal NZW rabbits (3.5 kg) to investigate effective stimulation parameters. The tibial nerve (TN) was surgically exposed, facilitating the implantation of a 2‐channel commercial cuff electrode (Nerve Cuff Electrodes; MicroProbes). An additional 2‐channel commercial cuff electrode (Nerve Cuff Electrodes; MicroProbes) for electrical stimulation was implanted in the proximal TN (distance: 27 mm) with a differential bipolar distance of 4 mm. Electrical stimulation was performed at 2 Hz for a total of 10 times for 5 s. Electrical stimulation parameters were a pulse width of 50 µs and interpulse delay of 10 µs. Stimulus intensities of 50, 200, and 400 µA were applied during recordings with a differential bipolar configuration. As a result of the recording, a compound nerve action potential (CNAP) was observed from the stimulus intensity of 200 µA, which showed an amplitude of 800 µV and a latency of 0.66 ms (Figure S5, Supporting Information). At the stimulation intensity of 400 µA, the amplitude of the CNAP was 5.8 mV and the latency was 0.65 ms, however, this stimulation induced a huge contraction of the muscle. These results match well with the previous study, showing the induced CNAP amplitude levels (hundreds of µV to several mV) in the sciatic nerve and its branches of rabbits.^[^ ^34^ ^]^ Based on this experiment, the range of stimulation intensity was safely determined from 10 to 300 µA for electrically evoking neural signals.

#### 2.2.2 Efferent Signal Recording with Bionic Interface (motor signal recording)

**Figure 3:**
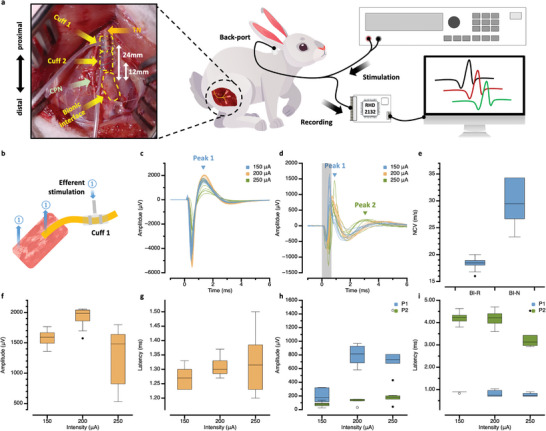
Recording characteristics of the evoked activity of the bionic interface in a rabbit model at 4 weeks. The bionic interface was formed by implanting a buckle interface after severing the nerve and transplanting a muscle graft to the distal end. The recording was performed through the back‐port connected to the bionic interface. a) Schematic illustration of the in vivo experiment and a photo of the bionic interface and commercial cuff electrodes implanted at 4 weeks for correlation analysis of nerve signals and RPNI signals from the BI‐N and BI‐R; CPN: common peroneal nerve. b) Schematic diagram of the stimulation and recording of efferent signals from the bionic interface. c) Induced efferent signals recorded at the BI‐R in the bionic interface. d) Induced nerve signals recorded at the BI‐N in the bionic interface and the stimulus artifact area of the neural signal are indicated by the gray box. e) Calculated NCVs of the first peak (P1) of the signals recorded at the BI‐R and BI‐N. f) Peak amplitude and g) latency changes of the signals recorded at the BI‐R with stimulus intensity changes. h) Peak amplitude and i) latency changes of the signals recorded at the BI‐N with stimulus intensity changes.

To evaluate the recording performance of the BI‐R and BI‐N at the bionic interface, electrically evoked efferent signals were recorded by each electrode after a stabilization period of 4 weeks at the bionic interface NZW rabbit model.^[^ ^26^ ^]^ After exposing the formed RPNI and nerve through an incision, a 2‐channel commercial cuff electrode (Nerve Cuff Electrodes; MicroProbes) was implanted on the proximal TN, 24 mm apart from the center of the bionic interface to stimulate the nerve (**Figure** **3**a). The electrical stimulation was performed at a strength of 150 to 250 µA. The induced efferent neural signals were recorded separately and simultaneously at the BI‐R (differential bipolar distance: 35 mm) and BI‐N (differential bipolar distance: 20 mm) with a neural signal acquisition process (Figure 3b). The detailed neural signal acquisition process is described in the Experimental Section.

When recording the induced efferent signals at the BI‐R, patterns were recorded for all 12 repetitions (Figure 3c). The shape and amplitude of the recorded signals have characteristics similar to those of typical RPNI signals.^[^ ^35^, ^36^ ^]^ The induced efferent signals at the BI‐N were also acquired for 12 stimuli, but sporadically different signals were observed (Figure S6, Supporting Information). The shape and peak latency (ms) of these sporadically recorded signals were very close to those of signals recorded at the BI‐R, but the amplitude was smaller. To verify that the other signals recorded at the BI‐N were electroneurography (ENG) signals, the sporadically recorded signals were removed from the signals recorded at the BI‐N, and all other signals were visualized (Figure 3d). Then, the recorded neural signals were observed after the stimulus artifact (the dark region in Figure 3d). Figure 3e shows the calculated nerve conduction velocity (NCV) based on the first peak (P1) of the signals recorded at the BI‐R and BI‐N. The NCVs were 18.49 ± 0.14 m ^−1^s for the BI‐R and 29.93 ±0.85 m ^−1^s for the BI‐N, indicating that the neural signal was faster than the RPNI signal. In the previous study, the NCV of 50 to 70 m ^−1^s and the muscle conduction velocity (MCV) of ≈35 m ^−1^s were reported in normal rabbits.^[^ ^37^, ^38^, ^39^ ^]^ These discrepancies are likely due to the incomplete recovery from the nerve damage and transection, which generally causes the reduction of NCV by ≈50%.^[^ ^40^, ^41^, ^42^, ^43^, ^44^ ^]^

Figure 3f shows the signal amplitudes recorded at the BI‐R depending on the stimulation intensity, indicating that the amplitude of the recorded signals increased with increasing stimulation intensity. Additionally, the amplitude of P1 averaged 0.8 mV at 200 µA stimulation, very similar to that of electrically evoked CNAP from the normal rabbits. However, the average latency was almost constant at 1.30 ms during the stimulation (Figure 3g), indicating that the RPNI signals were successfully recorded. Figure 3h shows the classified signal amplitudes of P1 and the second peak (P2) recorded at the BI‐N, depending on the stimulation intensity. The amplitude of the fastest signals (P1) increased with increasing stimulation intensity and was finally saturated, which is a typical response for motor fibers. The amplitude of relatively slow signals (P2) linearly increased with increasing stimulation intensity within the experimental intensity range, which is similar to the response of sensory fibers. Figure 3i shows the latency changes of P1 and P2, indicating that P1 is constant and lower (faster) than that recorded at the BI‐R, and P2 is larger (slower) than all and slightly reduced at the highest intensity. Furthermore, P2 appeared after the RPNI signal, implying that it was a sensory signal.^[^ ^45^ ^]^

The results show that electrically evoked RPNI and additional ENG signals can be obtained through the bionic interface under electrical stimulation. Additionally, acquiring nerve signals at the BI‐N allows the motor and sensory signals of the nerve to be obtained.

#### 2.2.3 Bidirectional Stimulation & Recording (Weeks 10, 15, 20)

**Figure 4:**
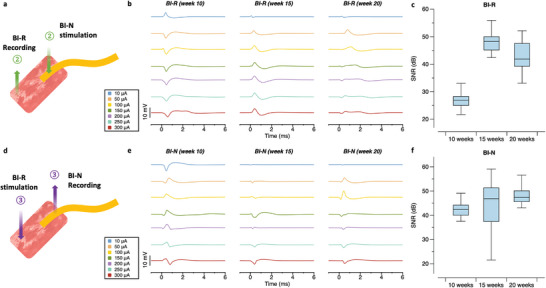
Recording and stimulation characteristics of the bionic interface in a rabbit model at 10, 15, and 20 weeks. The recording was performed through the back‐port connected to the bionic interface. a) Schematic diagram of the stimulation and recording of RPNI signals. b) RPNI signals recorded at the BI‐R according to the stimulation intensity in chronic implantation experiments from 10 to 20 weeks. c) SNR of RPNI signals recorded at the BI‐R in chronic implantation experiments. d) Schematic diagram of the stimulation and recording of induced neural signals. e) Signals recorded at the BI‐N according to the stimulation intensity in chronic implantation experiments from 10 to 20 weeks. f) SNR of signals recorded at the BI‐N in chronic implantation experiments.

After implantation, a time period of two months was selected as the stabilization and innervation period.^[^ ^26^ ^]^ Then, bidirectional stimulation and recording experiments using the implanted bionic interface were conducted every 5 weeks from 10 to 20 weeks after the surgery. The efferent RPNI signals evoked by BI‐N stimulation were recorded at the BI‐R (**Figure** **4**a), and the nerve signals induced by BI‐R stimulation were recorded at the BI‐N (Figure 4d). The electrical stimulation parameters were the same as those in the above experiments, but the intensity was increased from 10 to 300 µA.

The induced RPNI signals recorded at the BI‐R at 10 weeks showed an average latency of 0.454 ms and a maximum amplitude of 5.671 mV. Moreover, these values were 0.374 ms and 4.53 mV at 15 weeks. At 20 weeks, two peaks were observed for the recorded signal (Figure 4b). As the stimulation intensity increased, one peak gradually split into two peaks. The first peak had an average latency of 0.613 ms and a maximum amplitude of 0.952 mV. The second peak latency increased gradually from 0.8 to 2.7 ms with increased stimulation intensity, and the amplitude decreased from 4.652 to 1.942 mV. This tendency was reversible, i.e., the signals returned to their original state with decreasing stimulation intensity (Figure S7, Supporting Information). The results indicated that different groups of muscle fibers were activated according to the stimulation intensity.

The induced signals recorded at the BI‐N at 10 weeks showed an average latency of 0.5 ms and a maximum amplitude of 3.846 mV. Moreover, these values were 0.454 ms and 9.132 mV, respectively, at 15 weeks and 0.44 ms and 8.166 mV, respectively, at 20 weeks (Figure 4e). During the entire period, any multiple peaks (in the BI‐R stimulation) were not observed, indicating that nerve stimulation (BI‐N) could induce different muscle fiber contractions, while RPNI stimulation (BI‐R) could not. During all stimulations, twitching of the muscle graft was observed (Figure 4b,e).

The SNR was calculated for all recordings (Table S1, Supporting Information). For the bionic interface, the SNR of the RPNI signal recorded at the BI‐R was 26.74, 46.35, and 42.86 dB at 10, 15, and 20 weeks, respectively (Figure 4c), and the SNR of the signal recorded at the BI‐N at 10, 15, and 20 weeks was 42.37, 43.91, and 45.58 dB, respectively (Figure 4f). The SNR for the BI‐R dramatically increased at 15 weeks and stabilized afterward. This result indicates that stable BI‐R recording requires ≈15 weeks after the implantation, but not BI‐N recording. This result is in agreement with the fact that complete RPNI innervation requires more than 3 months^[^ ^46^ ^]^ where the BI‐R recordings may be affected by the RPNI innervation while the BI‐N recording on the nerve shows the stable SNR smoothly increased and saturated. There was a slight fluctuation of BI‐N SNR at 15 weeks, which might be the reason for the RPNI innervation but stabilized afterward. This result indicates that the complete innervation of the RPNI slightly affects BI‐N contact (between nerve and neural interface) but eventually stabilizes the contact for high‐quality recording. A slight reduction of the SNR of BI‐R at 20 weeks is due to the calculation selecting the highest peak among the split signals. The average SNR of all signals at 20 weeks was 33.0423 dB, and no significant performance degradation was observed during the implantation period. These results show that the SMP is a promising new material for neural interfaces for chronic implantation.

Furthermore, robotic leg control via electrically evoked BI‐R signals was demonstrated. Based on the threshold operation mechanism, the induced signals above the threshold operated well on the robotic leg (Table S2, Supporting Information). The detailed information is described in Figure S9 (Supporting Information), the Experimental Section, and the result and discussion in the supplementary information.

### 2.3 Naturally Evoked Signal Recording

**Figure 5:**
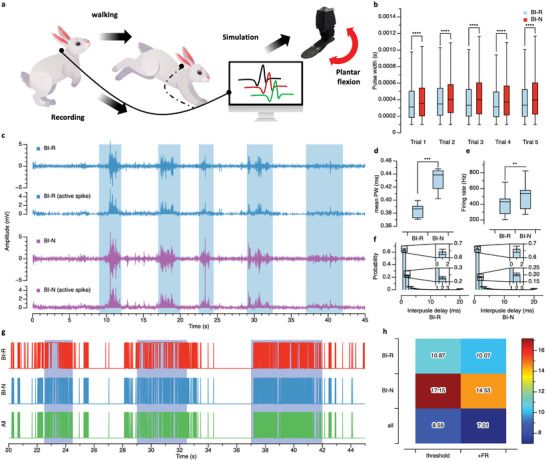
Recording characteristics of the bionic interface in an awake rabbit model and plantar flexion simulation of a robot operation based on the recorded signals. The recording was performed through the back‐port connected to the bionic interface. a) Schematic diagram of the naturally evoked somatic motor signal recording experiment and plantar flexion simulation using recording signal. b) Pulse widths (PWs) of all naturally evoked signals recorded at the BI‐R and BI‐N. c) Recorded naturally evoked BI‐R and BI‐N signals, and detected active spikes (> 3.5σ). The rabbit's walking phase is marked with a blue area. d) Mean PW and e) firing rate (FR) of the naturally evoked BI‐R and BI‐N signals. ISI of the naturally evoked neural signals. f) Mean ISI and firing rate of the naturally evoked neural signals. g) Robot drive signals (for plantar flexion) from neural prosthetic control during the simulation. The rabbit's walking phase is marked with a blue area. h) Error according to the input data during robot drive simulation;**p < 0.01, ***p < 0.001, ****p < 0.0001.

Naturally evoked somatic motor signals were recorded through an implanted back‐port while a rabbit walked (**Figure** **5**a). The recording configuration was the same as that in the above experiment, and the naturally evoked signals were simultaneously acquired at the BI‐R and BI‐N.

Walking was performed in a total of 5 trials. Active spikes were detected to confirm that the two types of somatic motor signals recorded through the bionic interface were distinguishable. These spikes were detected from the absolute signal value using a threshold of 3.5 times the root mean square (RMS) of the recorded signal noise and the threshold of the pulse width (0.1 ms) to remove the noise (Figure 5c).^[^ ^47^ ^]^ The pulse widths of all active spikes were measured, and an unpaired t‐test was performed on all pulse widths measured in the 5 trials. As a result, it was confirmed that there was a difference in pulse width between the two BI‐R and BI‐N signals during walking (Figure 5b). In addition, the average pulse widths of the BI‐R and BI‐N signals were compared for all trials, and as a result, it was confirmed that the average pulse width of the BI‐N signal was larger than that of the BI‐R signal (p = 0.0009) (Figure 5d). The interspike interval (ISI) histogram was investigated by measuring the interval between all active spikes, and the firing rate was measured for all trials (Figure 5e–f).^[^ ^48^, ^49^ ^]^ The mean firing rate was 413.20 Hz for the BI‐R and 510.73 Hz for the BI‐N (p = 0.0072), with a higher mean firing rate for the BI‐N. As a result, although recording the RPNI signal was inevitable due to the structure of the bionic interface in the BI‐N, the mean firing rate for the BI‐N was larger than that for the BI‐R because the nerve signal (ENG) was additionally recorded. In the ISI histogram, the average of the interpulse delay within 1 ms for BI‐R was 64.21%, the average for the interpulse delay within 2 ms was 22.83%, and for BI‐N, it was 66.18% and 20.35%. The reason why the probability of interpulse delay of less than 1 ms is higher in BI‐N may be due to the additional recording of neural signals, which underpin the results of neural signal acquisition through BI‐N recording. This result shows that the proposed bionic interface model enables the recording of different signals from the BI‐R and BI‐N when recording naturally evoked somatic motor signals, indicating that at least two different neural signals can be obtained from a single bionic interface.

A simple simulation of the plantar flexion movement performed by the gastrocnemius muscle, controlled by the TN, was performed to investigate the potential of these signals for controlling bionic limbs. The naturally evoked somatic motor signals were recorded while a rabbit walked for 45 s, and the video was recorded simultaneously (Video S1, Supporting Information). Using the recorded video, the signals evoked while walking and the signals evoked without walking were labeled. The recorded signals from 0 to 20 s were set as the training area, and the recorded signals from 20 to 45 s were set as the test area. Then, within the training area, the average firing rate of the active spikes measured in the labeled walking area was set as the threshold. The simulation was designed to output a robotic motion signal when the firing rate of active spikes in one window was higher than the threshold (Figure 5g). The size of the window for one action (plantar flexion) was 150 samples (5 ms), and the window delay was 30 samples (1 ms). The simulation results showed that when active spikes were detected and the plantar flexion movement was simulated, the error was 10.87% when driven only by the motor RPNI signals and 17.15% when driven only by the motor nerve signals. Interestingly, when both the RPNI and nerve signals were used, the error was 8.59%. Additionally, the average firing rate threshold was added as a condition for plantar flexion simulation, and the errors were 10.07% when driven by only the motor RPNI signals, 14.53% when driven by only the nerve signals, and 7.01% when both signals were used (Figure 5h). These results confirm that it is more advantageous in terms of the error to form a bionic interface and record both somatic motor nerve signals and RPNI signals to control the neural prosthetic device rather than controlling it only with RPNI signals.

### 2.4 Histological Test

**Figure 6:**
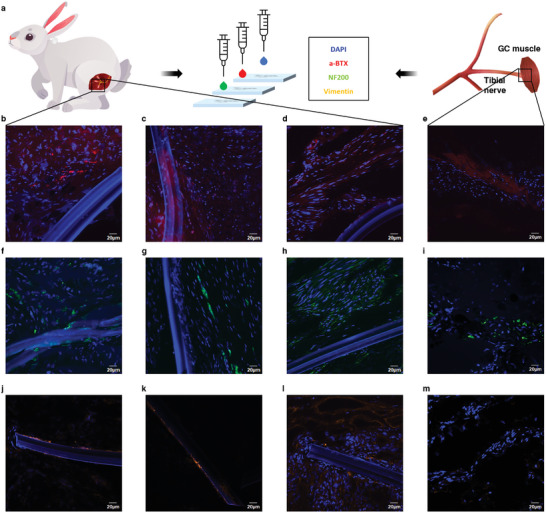
Histological examination for innervation validation and measurement of fibrosis a) Schematic diagram of the immunostaining of the bionic interface. b–d) a‐BTX and f–h) NF200 staining of the interface‐hybrid RPNI sample at 29 weeks. e) a‐BTX and i) NF200 staining results of the normal NMJ. j–l) Vimentin staining results around the buckle interface and m) the normal NMJ.

Rabbits were euthanized at week 29, and biopsies were performed at the bionic interface. Alpha‐bungarotoxin (a‐BTX) and neurofilament 200 (NF200) immunohistochemical staining was performed on each RPNI tissue sample and normal neuromuscular junction (NMJ) to label the postsynaptic acetylcholine receptor, which is used to evaluate the regeneration and reinnervation of NMJs (**Figure** **6**a).^[^ ^50^, ^51^, ^52^ ^]^ Upon a‐BTX and NF200 immunostaining, colocalization of a‐BTX and NF200 was identified in RPNI tissue (Figure 6b–d,f–h), and the size of the expression area was similar to that in normal neuromuscular tissue (Figure 6e,i). This finding confirms the new formation of acetylcholine receptors in the RPNI, which indicates the regeneration of NMJs at the nerve interface.

In addition, vimentin staining was performed to determine the biocompatibility of the chronically implanted buckle interface. High vimentin expression suggests myofibroblast proliferation, which indicates the degree of fibrosis. Vimentin expression was confirmed around the interface (depth: 7.99 µm) (Figure 6j–l). There was little vimentin expression except for some fibrosis around the neural interface, which was comparable to that of the normal NMJ (Figure 6m). The formation of less fibrosis may contribute to high‐quality signal (high SNR) acquisition.^[^ ^53^, ^54^, ^55^ ^]^ These histological examination results show that the bionic interface is successfully reinnervated with regenerated NMJs despite the implantation of the neural interface inside the RPNI. Additionally, it shows the superiority of the SMP used as a material for fabricating the neural interface for chronic transplantation.

## 3 Conclusion

We propose a novel hybrid bionic interface that combines a biological interface with a peripheral nerve interface to enhance the diversity of recording and stimulation capabilities by achieving simultaneous nerve and muscle graft contact with a single interface. As proof‐of‐concept, we demonstrated that a buckle‐shaped nerve interface using a SMP could be easily and quickly implanted on a severed nerve. Through 29 weeks of chronic implantation experiments in a rabbit model, we confirmed the potential of the SMP‐based neural interface for stable chronic implantation and for stimulation and signal recording at the muscle graft and the nerve tissues inside the RPNI. It was also confirmed that the different signals could be recorded and separated via BI‐R and BI‐N electrodes, even though the nerve interface was wrapped with muscle tissue. The implanted neural interface also recorded a slow peak of sensory neural signals, which could possibly be useful for sensory recording applications. One of the interesting results is that the BI‐N stimulation induces the split multiple peaks of the RPNI at 20 weeks after the stabilization, but this could not be observed during the whole BI‐R stimulation. This shows the possibility that the formation of this hybrid bionic nerve interface induces split RPNI recording, providing much information corresponding to different efferent signals, as well as, it provides different stimulation roles like various sensory feedbacks via RPNI stimulation and nerve stimulation.

Moreover, the SNR results of the implantation during 10, 15, and 20 weeks indicate that stable RPNI recordings by BI‐R require ≈15 weeks, which matches well with the complete innervation time of RPNI (>3 months). Also, the formation of RPNI slightly caused the fluctuation of the SNR from the BI‐N recording but overall SNR increased and saturated. The bionic interface was able to record high‐quality signals (>30 dB), which was expected due to the firm embedding of the implanted neural interface within the muscle tissue.

Furthermore, we demonstrated the potential of this bionic interface for neuroprosthetic applications by successfully acquiring somatic motor signals during the natural walking of rabbits using the two channels. These results indicate that two different signals can be acquired from the bionic interface, and increasing the number of obtainable neural signals via the bionic interface can improve the accuracy of robot control through a simple simulation.

In terms of chronic implantation, the results confirmed that the SMP is a promising material for fabricating neural interfaces for chronic implantation. The superiority of the buckle interface made using the SMP was confirmed by observing successful innervation even when the neural interface was implanted inside the RPNI, indicating that the bionic interface could serve as a new peripheral nerve interface. Although the advantage of acquiring additional nerve signals was confirmed by forming our bionic interface, there is a limit to acquiring more detailed nerve signals because it is in the form of an extra‐neural electrode like a cuff. Forms of intra‐neural or regenerative approaches where electrodes are positioned inside a nerve would be preferable to maximize the performance of recording and stimulation in fascicles or even fiber levels. In this aspect, we expect advanced hybrid bionic nerve interface technology continuously being developed could have a significant effect on intuitive and perceptual bionic limbs in the future.

## 4 Experimental Section

### Material Synthesis

1,3,5‐Triallyl‐1,3,5‐triazine‐2,4,6(1H,3H,5H)‐trione (TATATO) (Sigma‒Aldrich Korea), trimethylolpropanetris(3‐mercaptopropionate) (TMTMP) (Sigma‒Aldrich Korea), tricyclodecanedimethanoldiacrylate (TCMDA) (Sigma‒Aldrich Korea), and 2,2‐dimethoxy‐2‐phenyl‐acetophenone (DMPA) (Sigma‒Aldrich Korea) were used to synthesize the SMP. The synthesis process was conducted in a class 1000 clean room. To obtain a uniform SMP solution, the SMP was mixed as reported in previous studies.

### Interface Design and Fabrication

The buckle interface was designed in consideration of the need for bidirectional communication. By mimicking the buckle strap, stimulation, and recording could be performed on both the inner area of the electrode (BI‐N; nerve contact area) as well as the outer area of the electrode (BI‐R; muscle contact part) while the electrode was wrapped around the nerve. The developed buckle design enables easy and robust implantation through fast buckling during surgery (Figure 1). The interface was fabricated using a standard photolithography process, and further information about the fabrication process could be found in previous studies.^[^ ^29^ ^]^ To fabricate the 1st SMP layer, the SMP solution was spin‐coated (800 rpm, 25 s) on a wafer coated with 1 µm of aluminum (Al). The SMP was polymerized for 30 min under UV radiation at a wavelength of 365 nm. Afterward, it was cured for 24 hours in a vacuum oven at 120 °C. Additionally, a photoresist (DNR‐L300‐40, Dongin‐Semichem) for patterning the metal layer was spin‐coated and then patterned through a mask aligner. Afterward, chrome (Cr; 50 nm) and gold (Au; 200 nm) were sputtered, and the metal layer was patterned through the removal of the photoresist. The 2nd SMP layer was coated and cured in the same way as before. As a hard mask for etching, Cr (150 nm) was patterned in the same way as the metal layer. The reactive ion etching of the SMP was carried out using O_2_ plasma. The Cr hard mask was removed by Cr etchant. The interface was released by removing the Al through an Al etchant (Figure S1a, Supporting Information).

### Interface Packaging

The manufactured interface was packaged to be biocompatible and stretchable for stable long‐term implantation. With this packaging, the electrode not only was completely encapsulated but also had some elasticity to protect it from the tension applied to the wire in vivo. First, to wire the electrodes of each channel without interference, a custom flexible printed circuit board (FPCB) pad was bonded using anisotropic conductive film (ACF) at 5 MPa at 180 °C for 10 s (Figure S1b, Supporting Information). To connect the back‐port (P1 technology) and the neural interface, an implantable wire (DFT wire, Fort Wayne Metals) was fixed in a helical structure within a 65 cm stretchable and biocompatible PVC tube (Figure S1c, Supporting Information). The back‐port and buckle interface were fixed at both ends of the stretchable wire through soldering. The soldered part was encapsulated in medical epoxy (LOCTITE EA M‐121HP, Henkel) (Figure S1d, Supporting Information). The samples with the manufactured helical structure wire were able to withstand a maximum strain of 110% without tearing and impedance change (Figure S2, Supporting Information).

### IrO_2_ Coating

All channels of the buckle interface were coated with iridium oxide (IrO_2_) to improve the electrode performance and ensure stable chronic implantation. To prepare the IrO_2_ coating solution, 300 mg of iridium chloride was added to 200 ml of DI water and stirred for 15 min. Oxalic acid powder (1000 mg) was added to the solution and stirred for 10 min. Potassium carbonate was used to control the pH of the solution to 10.5. The prepared solution was rested at room temperature for 2 days, during which it became dark blue. IrO_2_ was electrodeposited through a multichannel potentiostat (Ivium‐n‐Stat; IVIUM Technology). Only the counter electrode and working electrode were used for IrO_2_ coating. A platinum mesh electrode was used as a counter electrode, and a fabricated neural interface was used as a working electrode. A voltage of 0 to 0.76 V was applied at a scan rate of 50 mV s^−1^, with 50 repeats (Figure S3a, Supporting Information).

### Interface Characterization

The performance of the buckle interface was investigated using electrochemical impedance spectroscopy (EIS) with a multichannel potentiostat (Ivium‐n‐Stat, IVIUM Technology). A three‐electrode configuration with a silver/silver chloride (Ag/AgCl) electrode as the reference electrode, a platinum (Pt) electrode as the counter electrode, and phosphate‐buffered saline (PBS) as the medium was used. Impedance was measured in the frequency range of 1 Hz to 100 kHz, and the CV curve was measured at a scan rate of 50 mV s^−1^ in a window of −0.6 to 0.8 V. The electrode size of the buckle interface was 0.5026 mm^2^, and the CSC was calculated from the measured CV curve according to Equation (1).^[^ ^56^ ^]^

(1) $$\mathrm{CSC}\;=\;\frac{\mathrm{area}\;\mathrm{of}\;\mathrm{CV}\;\mathrm{curve}}{\mathrm{electrode}\;\mathrm{surface}\mathrm{area}\;\times \;\mathrm{scanning}\;\mathrm{rate}}$$

### Ethics Statement

The animal care and use protocol were reviewed and approved by the Institutional Animal Care and Use Committee at Daegu Gyeonbuk Institute of Science and Technology (Approval No. DGIST‐IACUC‐21012702‐0001) and the Institutional Animal Care and Use Committee of Asan Institute for Life Sciences (Approval No. 2022‐12‐071 and 2022‐13‐072).

### Animals & Surgical Procedure

New Zealand White (NZW) rabbits with a weight range of 3.0 to 3.3 kg were used for the experiment. To anesthetize the animals, 0.2 ml kg^−1^ alfaxalone and 0.1 ml kg^−1^ rompun were administered via intramuscular injection. The animals were then further anesthetized with vaporized isoflurane (2.5%) in a constant oxygen flux. A 3 cm incision was made in the femur to show the bicep femoris and vastus lateralis. The bicep femoris and vastus lateralis were separated and fixed with a retractor so that the TN was sufficiently visible (Figure S4a, Supporting Information). The bicep femoris muscle was sliced thinly in a rectangular shape of 3 cm × 2 cm (Figure S4b, Supporting Information). To form the bionic interface, the TN was severed (Figure S4c, Supporting Information), and a buckle interface was implanted on the severed nerve (Figure S4d, Supporting Information) and wrapped with the sliced muscle (Figure S4e, Supporting Information). For back‐port tunneling, two incisions of ≈1.5 cm were made on the back and neck. The back‐port was tunneled subcutaneously to the incision in the back of the neck. The back‐port was connected to the back‐mount (P1 technology) and sutured and fixed to the incision in the back of the neck. To ensure the long‐term stability of the back‐port during chronic implantation, the suture was encapsulated with dental cement (Figure S4f, Supporting Information). The operation was completed after all incisions were sutured. Afterward, 0.1 ml kg^−1^ tramadol and 0.2 ml kg^−1^ enrofloxacin were intramuscularly injected twice a day for 2 weeks, and the state of the rabbit was observed. The rabbits were divided into two groups for experiments, and in the first group, after the experiment in Section 2.2.2 (after 4 weeks of implantation), euthanasia was performed by inhalation of high‐concentration vaporized isoflurane under anesthesia. The second group was euthanized in the same way after the experiments in Sections 2.2.3 and 2.3 (after 29 weeks of implantation) had been completed.

### Investigation of Stimulation Parameters for Neural Signal Acquisition

To investigate the electrophysiological properties of TN in normal NZW rabbits, a 2‐channel commercial cuff electrode (Nerve Cuff Electrodes; MicroProbes) was implanted in the TN for electrically evoked CNAP recording, and an additional 2‐channel commercial cuff electrode (Nerve Cuff Electrodes; MicroProbes) for electrical stimulation was implanted in the proximal TN (distance: 27 mm). A stainless‐steel wire was implanted into the nearby muscle as a ground electrode for recording. Recordings were performed in a differential bipolar configuration (differential bipolar distance: 4 mm). Among the two channels of the cuff electrode (for recording), the proximal electrode was used as the working electrode and the distal electrode was used as the reference electrode. Differential biopolar recording was performed at a 100 k sampling rate, and a Data Acquisition system (PowerLab 8/35; ADInstruments) and pre‐amplifier (SR560; Stanford Research System) were used. A 60 Hz notch filter was applied to remove harmonic noise.

### Optimization of Stimulation Parameters for Neural Activation

The stimulation parameters for activating the damaged and regenerated TN were investigated through TN stimulation and CNAP recording through a buckle interface implanted on the RPNI formed at the TN. Differential bipolar ENG signals were acquired by the neural signal acquisition process. As a result, from the stimulation of 150 µA, the ENG signal was recorded and overlapped with the stimulus artifact. As the stimulation intensity increased, electrically evoked neural signals could be recorded up to a stimulus intensity of 250 µA (Figure S6, Supporting Information). As a result, all neural and RPNI signals were electrically evoked within the stimulation parameter range from 150 to 250 µA.

### Neural Signal Acquisition

To protect the electrode and tissue from electrical stimulation charge, stimulation was performed using biphasic pulses.^[^ ^57^, ^58^ ^]^ Electrical stimulation was performed 12 times for 1 min at 0.2 Hz. The electrical stimulation parameters were a 50 µs pulse width and a 10 µs interpulse delay. Each channel of the neural interface was used as the working electrode, and a stainless wire implanted subcutaneously on the back was used as the reference electrode. A stainless wire that functioned as a ground electrode was attached to the rabbit's ear. The bipolar recording was performed at a 30 k sampling rate for each channel of the bionic interface through an electrophysiology amplifier (RHD 2312; Intan Technology) then subtracted each pair for a differential bipolar recording via a Matlab (Figure S8, Supporting Information). The recorded signal was notch‐filtered at 60 Hz to remove harmonic noise and passed through a second‐order Butterworth high‐pass filter with a cutoff frequency of 300 Hz and a second‐order Butterworth low‐pass filter with a cutoff frequency of 3500 Hz. A differential bipolar recording method was applied for neural signals recorded from each channel through a Matlab to acquire the low‐noise neural signals for correlation analysis.

### SNR Calculation

SNRs were calculated for all recorded signals. The RMS value was calculated with the signal epoch up to 6 ms after stimulation. The RMS value of the noise recorded from 1 s to 6 ms after stimulation was calculated. The SNR was calculated with Equation 2 with the calculated RMS of the signal epoch and the RMS of the noise epoch.

(2) $$\mathrm{SNR}\left(\mathrm{dB}\right)\;=\;20\log\frac{{\mathrm{rms}}_{\mathrm{signal}\;\mathrm{epoch}}}{{\mathrm{rms}}_{\mathrm{noise}\;\mathrm{epoch}}}$$

### Error Calculation

The error of the simulation results was derived by calculating how much the robot operated in areas where the rabbit did not walk. The number of samples for which the robot did not operate in the nonwalking area (TN: true negative) and the number of samples for which the robot operated in the nonwalking area (FP: false positive) were measured, and the error was calculated using Equation (3).

(3) $$\mathrm{Error}\;\left(\%\right)\;=\frac{FP}{TN+FP}\;$$

### Immunofluorescence Staining for Histological Testing

Bionic interfaces formed through buckle interface implantation were isolated 4 and 29 weeks after implantation and evaluated histologically using immunofluorescence staining. The collected tissue was rapidly frozen in liquid nitrogen at −196 °C after being embedded in an optimal cutting temperature (OCT) compound. For staining, 10 µm cryosections of each tissue were air‐dried. The size of cryosected tissues varied from 10 mm × 10 mm to 20 mm × 20 mm.

After harvest, tissues were placed in 4% paraformaldehyde solution (Electron Microscopy Science, Hatfield, Pennsylvania) in PBS for 10 min at room temperature. After whole‐tissue fixation, all tissue slides were washed 3 times for 5 min in PBS and incubated in PBST (0.1% Triton X‐100 in PBS) for 10 min at room temperature. Then, the slides were again washed 3 times for 5 min in PBS. The tissues were stained with bungarotoxin antibodies. Two hundred microliters of diluted rabbit anti–bungarotoxin (1:200; Thermo Fisher, Eugene, Oregon) in antibody diluent (ScyTek) was applied per slide, and the slides were then incubated for 1 h at 37 °C, followed by washing 3 times for 5 min in PBS. Finally, the slides were mounted by applying several drops of mounting medium with DAPI (Golden Bridge International, USA) and a coverslip.

Vimentin immunohistochemistry staining was performed as follows. After whole‐tissue fixation with 4% paraformaldehyde solution, all tissue slides were washed 3 times for 5 min in PBS, incubated in PBST (0.1% Triton X‐100 in PBS) for 10 min at room temperature and again washed 3 times for 5 min in PBS. The tissues were then stained with 200 µl of diluted Vimentin antibody (1:200; Santa Cruz Biotechnology, Heidelberg Germany) in antibody diluent (ScyTek) and incubated overnight at 4 °C. The slides were then washed 3 times for 5 min in PBS. Then, antibody enhancer (Polink‐2 Plus Mouse HRP kits, OriGene) was applied at 200 µl per slide, followed by incubation for 30 min at room temperature. Afterward, polymer (Polink‐2 Plus Mouse HRP kits, OriGene) was applied at 200 µl per slide, followed by incubation for 30 min at room temperature. The slides were then washed 3 times for 5 min in PBS, and color development DAB (ScyTek) was applied for 5 min, followed by washing with tap water. Counterstaining with 1 ml or a sufficient volume of hematoxylin (BBC Biochemical) was performed by completely covering the slide tissue with the stain and waiting for ≈45 seconds. Slides were then placed in 1X TBST until they turned blue (≈30–60 s) and then rinsed well in distilled or tap water. In turn, the slides were placed in 95% and 100% ethanol for 10 s and then dipped in xylene for 10 s. Finally, the slides were mounted by applying several drops of permanent‐mount (Acrymount, StatLab) and a coverslip.

For a‐BTX and NF200 immunohistochemistry staining, RPNI tissue was placed in 4% paraformaldehyde in PBS for 10 min at room temperature. All tissues were washed 3 times for 5 min in PBS, incubated in PBST (0.1% Triton X‐100 in PBS) for 10 min at room temperature, and washed 3 times for 5 min in PBS. The tissues were then stained with diluted NF200 antibody (1:200; MBS, San Diego) or a‐BTX antibody (1:200; Thermo Fisher, Eugene, Oregon) in antibody diluent (ScyTek) at 200 µl per slide. Then, the slides were incubated overnight at 4 °C. Antibody enhancer (Polink‐2 Plus Mouse HRP kits, OriGene) was applied at 200 µl per slide, followed by incubation for 30 min at room temperature. Afterward, polymer (Polink‐2 Plus Mouse HRP kits, OriGene) was applied at 200 µl per slide, followed by incubation for 30 min at room temperature. Color development DAB (ScyTek) was applied for 5 min. After washing the slides in tap water, they were washed in distilled water for 1 min. Counterstaining with 1 ml or a sufficient volume of hematoxylin (BBC Biochemical) was performed by completely covering the slide tissue with the stain and waiting for ≈45 s, followed by rinsing well in tap water for 1–2 min. The slides were then placed in 1X TBST until they turned blue (≈30–60 s) and then rinsed well with distilled or tap water. In turn, the slides were alternately placed in 95% and 100% ethanol for 10 s and then dipped in xylene for 10 s. Finally, the slides were mounted by applying several drops of permanent‐mount (Acrymount, StatLab) and a coverslip.

## Conflict of Interest

The authors declare no conflict of interest.

## Supporting information

Supporting Information

Supplemental Video 1

## Data Availability

The data that support the findings of this study are available from the corresponding author upon reasonable request.
