# Supplementary material for: Hybrid Bionic Nerve Interface for Application in Bionic Limbs
Source: Adv Sci (Weinh). 2023 Oct 15;10(35):2303728. doi: 10.1002/advs.202303728 (PMC10724394; doi:10.1002/advs.202303728)
Supplement: Supplementary file 1 — Supporting Information [file ADVS-10-2303728-s001.pdf]

ADVANCED SCIENCE
Open Access

Supporting Information

for *Adv. Sci.*, DOI 10.1002/advs.202303728

Hybrid Bionic Nerve Interface for Application in Bionic Limbs

Youngjun Cho, Hyung Hwa Jeong, Heejae Shin, Changsik John Pak, Jeongmok Cho, Yongwoo Kim, Donggeon Kim, Taehyeon Kim, Hoijun Kim, Sohee Kim, Soonchul Kwon, Joon Pio Hong, Hyunsuk Peter Suh and Sanghoon Lee**

Supporting Information

Hybrid bionic nerve interface for application in bionic limbs

Youngjun Cho[+]*, HyungHwa Jeong*[+]*, Heejae Shin, ChangSik John Pak, Jeongmok Jo, YongWoo Kim, Donggeon Kim, Taehyeon Kim, Hoejun Kim, Sohee Kim, Suncheol Kwon, Joon Pio Hong, HyunSuk Peter Suh*[*] *and Sanghoon Lee*[*]

Y. Cho, H. Shin, Y. Kim, Prof. S. Kim, Prof. S. Lee
Daegu Gyeongbuk Institute of Science and Technology (DGIST), Department of Robotics and Mechatronics Engineering, Daegu, 42899, Korea
E-mail: hoonw@dgist.ac.kr

H. Jeong, Prof. C. J. Pak, J. Jo, D. Kim, T. Kim, Prof. J. P. Hong, Prof. H. P. Suh
Asan Medical Center, Department of Plastic and Reconstructive Surgery, Seoul, 05505, Korea

H. Kim, Prof. S. Kwon
Department of Smart Convergence, Kwangwoon University, Seoul, 01890, Korea

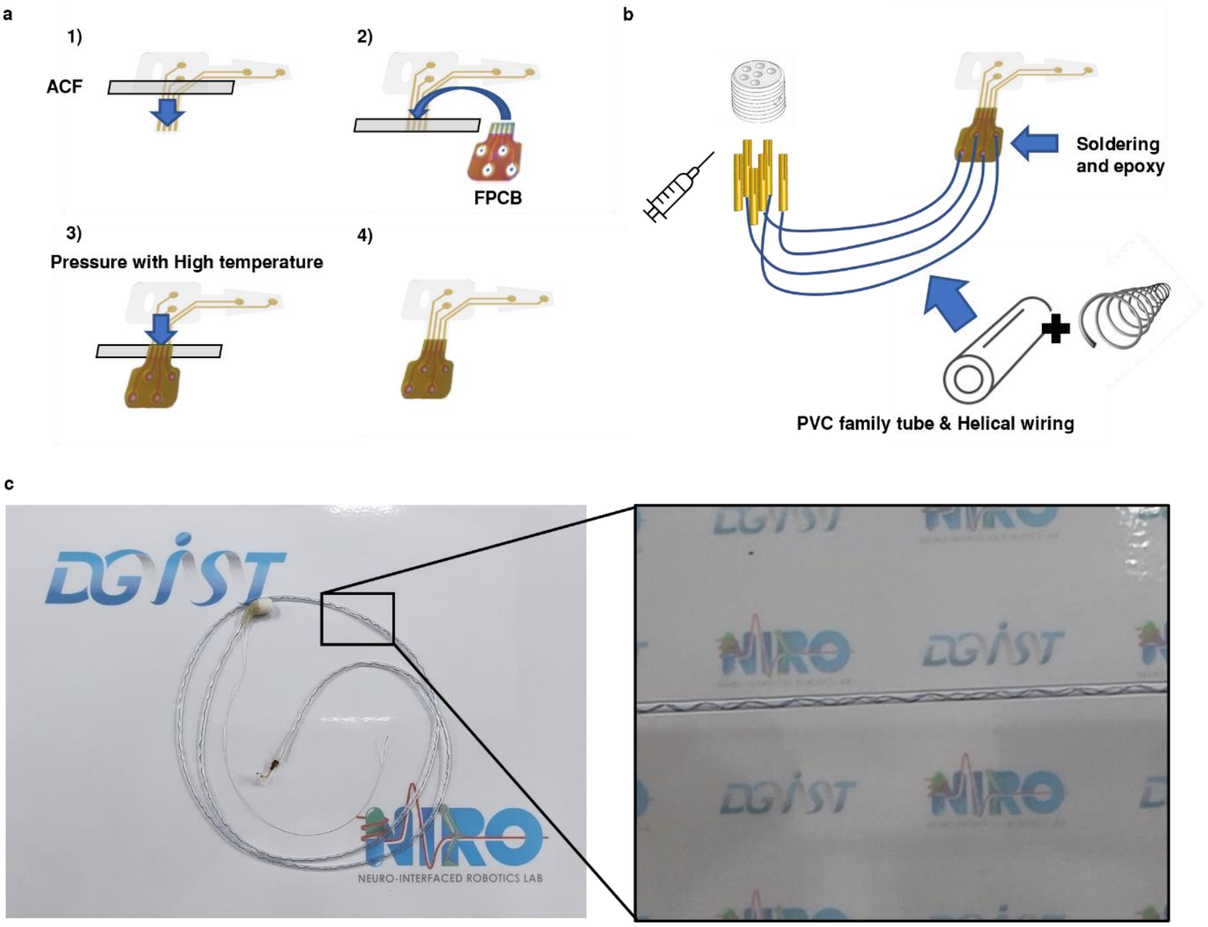

Figure S1. Buckle interface packaging. (a) Schematic diagram of ACF bonding for wiring the buckle interface. (b) Schematic diagram of wire packaging for head-port connection. For data acquisition during chronic implantation, a 65 cm long wire was connected to the head-port and buckle interface. To ensure durability during chronic implantation, the wire was packaged in a helical structure to withstand tension. (c) Completed buckle interface package and wire with helical core structure.

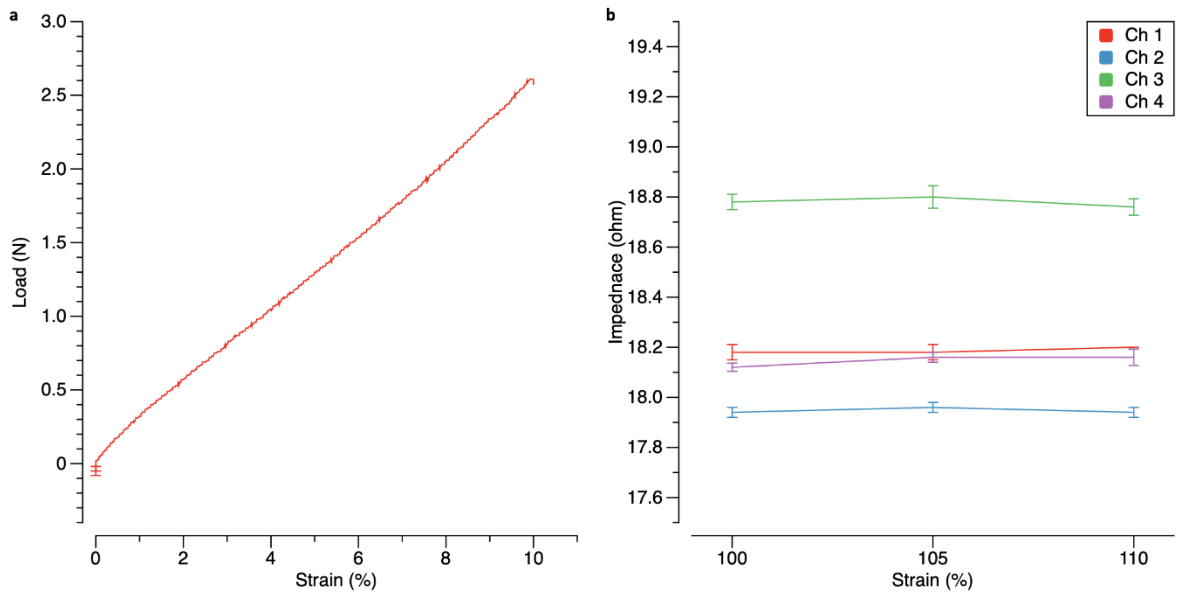

Figure S2. Result of the packaged wire strain test. (a) Tensile strength test and (b) strain-impedance test of the packaged wire. The packaged wire was stretched up to 110% through a tensile compression stand (MultiTest-dv; Mecmesin). Impedance measurement of each channel of the packaged wire for each tensile rate showed stable impedance even at 110% tensile rate.

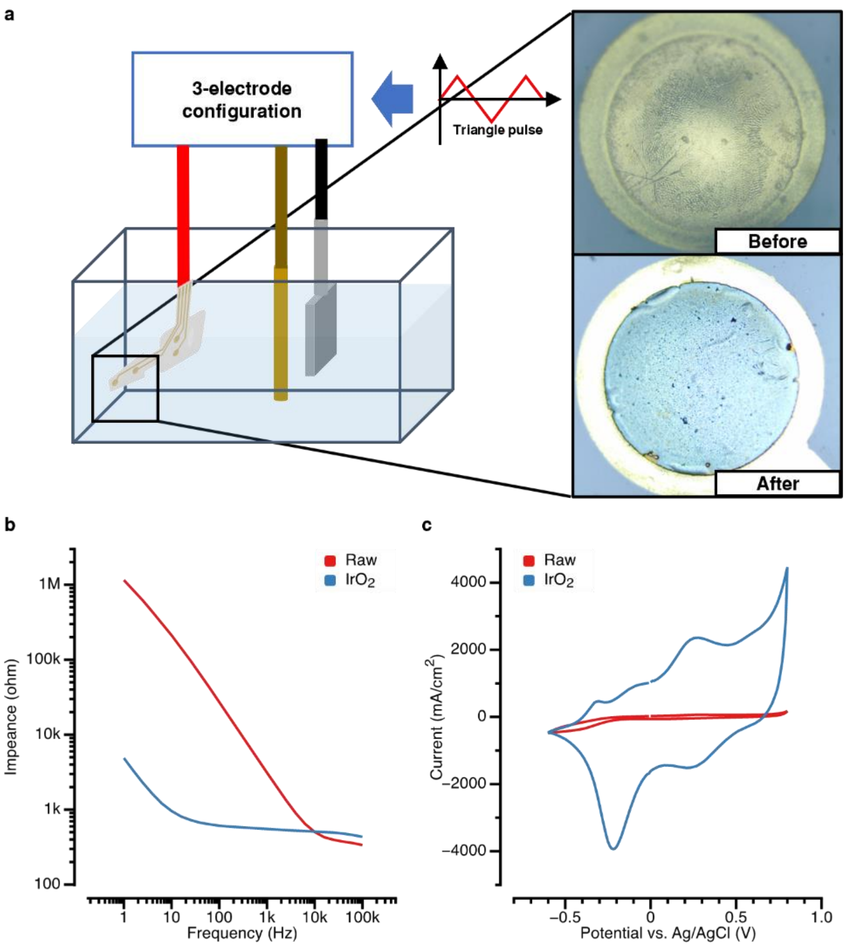

Figure S3. Iridium oxide (IrO_2) coating. (a) Schematic diagram of the iridium oxide coating method and comparison of electrodes before and after coating. (b) Impedance changes after IrO_2 coating. (c) CSC change after IrO_2 coating.

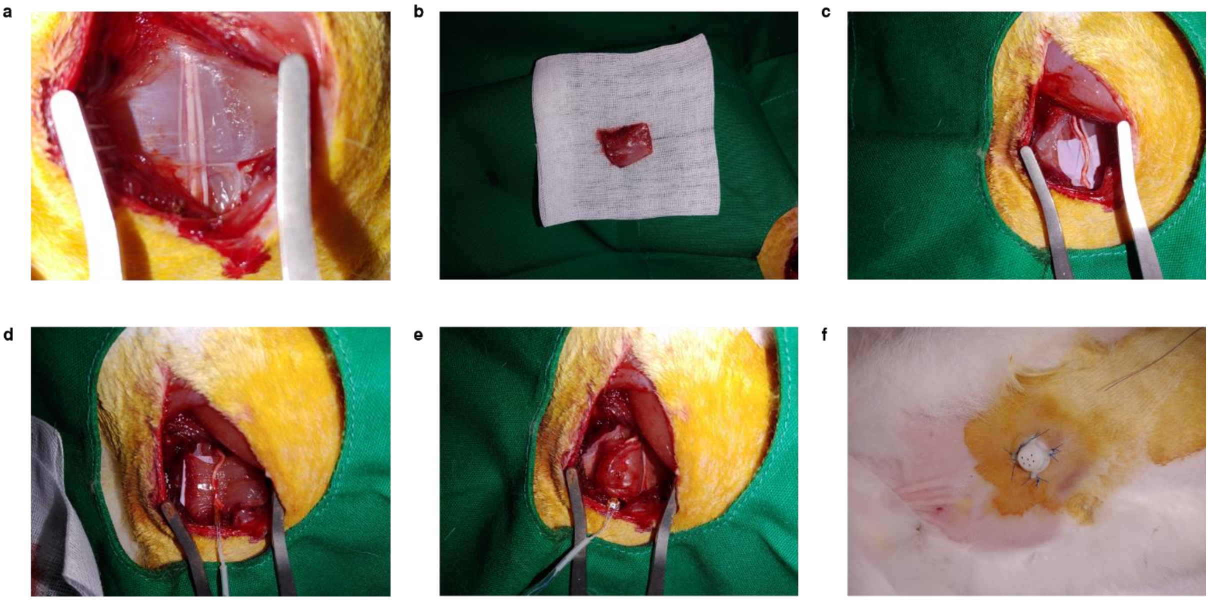

Figure S4. Surgical procedure for the formation of a hybrid bionic nerve interface.

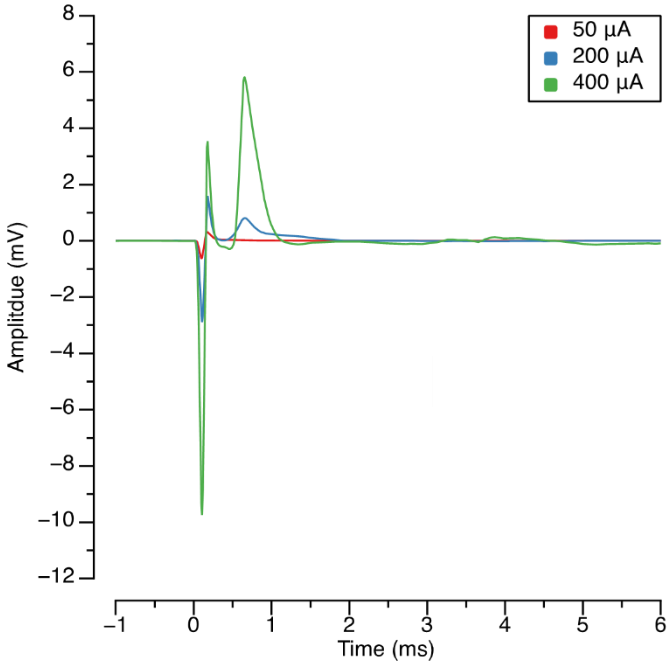

Figure S5. Results of electrophysiological recordings by electrical stimulation at normal rabbits. Electrically evoked CNAP recordings for stimulation intensities of 50 µA, 200 µA, and 400 µA.

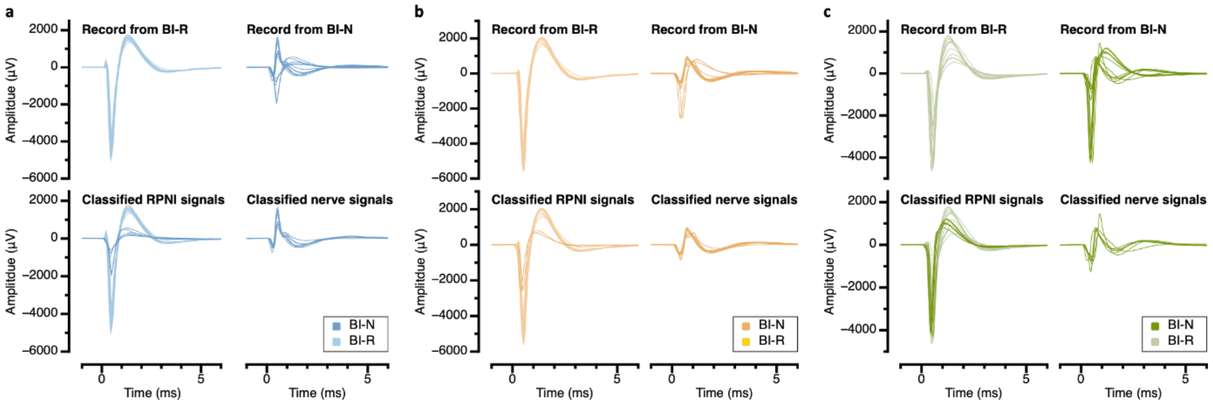

Figure S6. Results of 4-week efferent signal acquisition and classification of RPNI and nerve signals. Neural signals and RPNI signals were separated based on the number of peaks that could be specified excluding stimulus artifacts (number of peaks in the RPNI signal: 1) number of peaks in the neural signal: 2) and amplitude. (a) 150 μA. (b) 200 μA. (c) 250 μA.

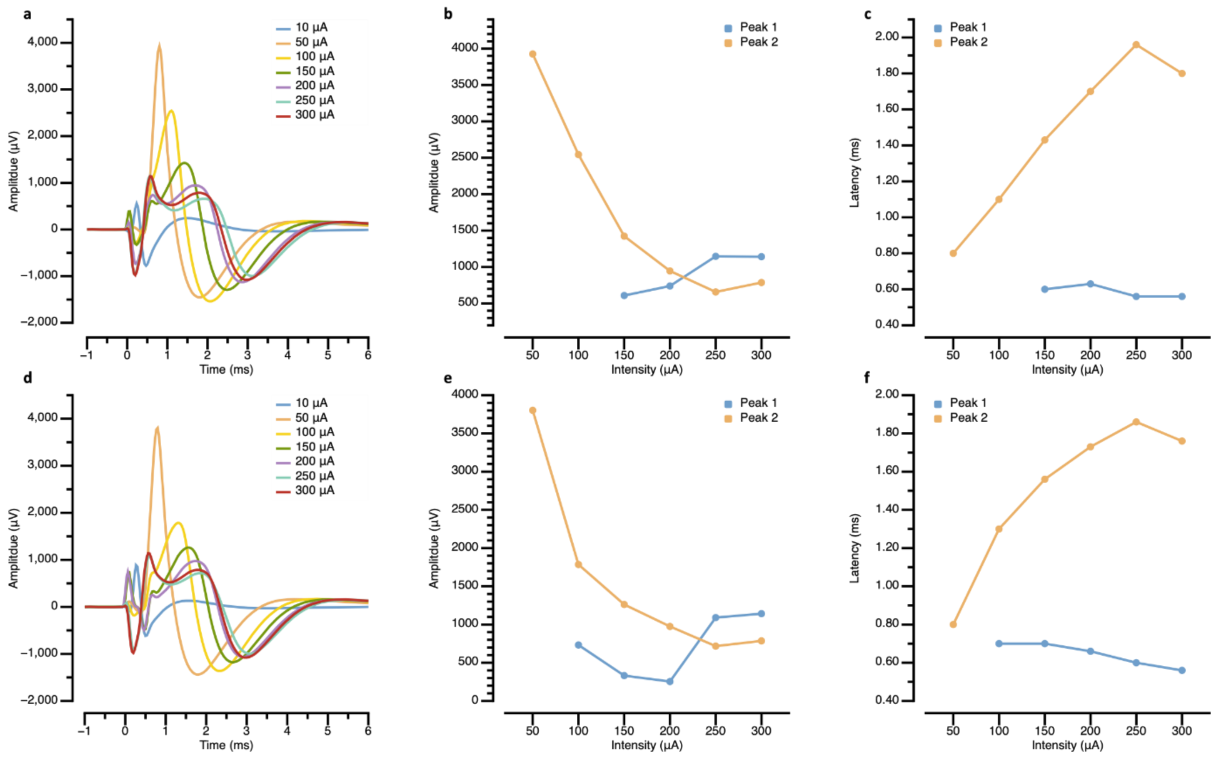

Figure S7. BI-R neural signal according to the stimulation intensity recorded at 20 weeks after implantation. (a) Signals recorded at the BI-R evoked by increasing the stimulation intensity from 10 μA to 300 μA. (b) Amplitude change according to the stimulus intensity. (c) Latency changes according to the stimulus intensity. (d) Signals recorded at the BI-R evoked by decreasing the stimulation intensity from 300 μA to 10 μA. (e) Amplitude change according to the stimulus intensity. (f) Latency changes according to the stimulus intensity.

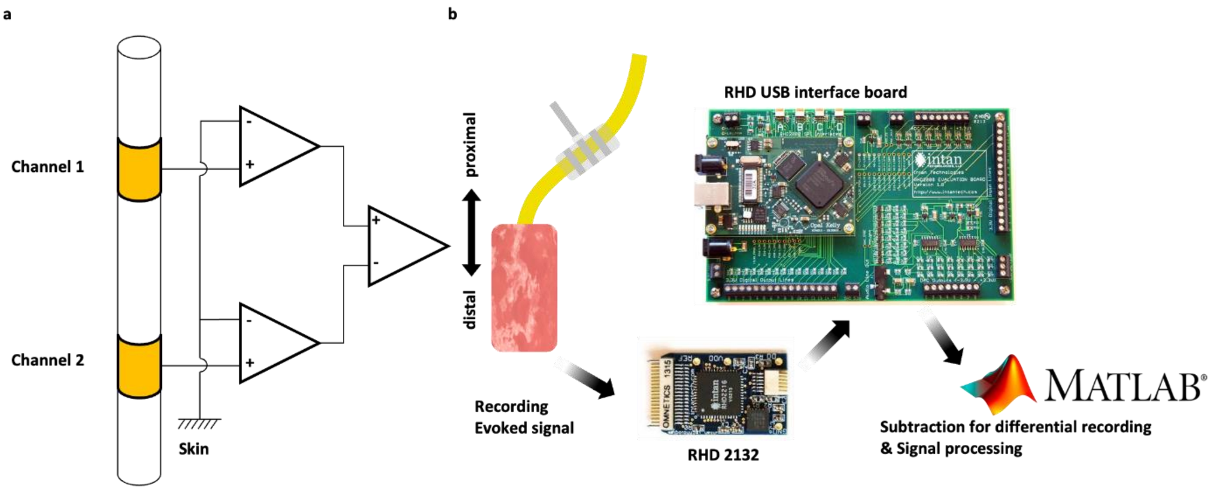

Figure S8. Recording configuration for neural signal acquisition. Electrically evoked neural signals were induced by stimulation through cuff electrodes placed on the proximal nerve of the RPNI. (a) Recording configuration for differential bipolar signal acquisition. (b) Schematic diagram of neural signal acquisition.

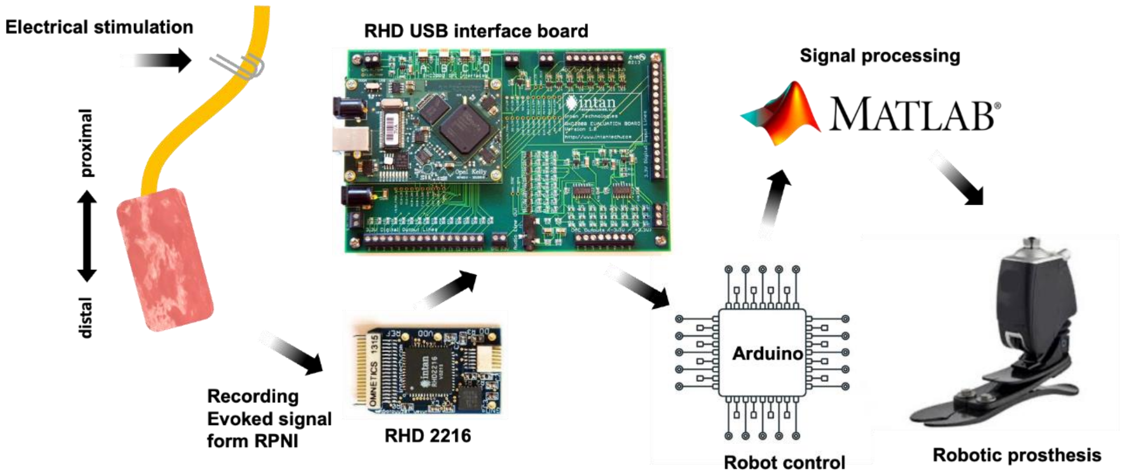

Figure S9. Schematic diagram of neural prosthesis control through electrically evoked BI-R signals.

Table S1. SNR of neural signals evoked from chronic implantation.

Stimulus Intensity (μA)	Week 10 (dB)		Week 15 (dB)		Week 20 (dB)	
	BI-R	BI-N	BI-R	BI-N	BI-R	BI-N
**10**	26.81	47.12	31.99	23.91	36.23	28.45
**50**	26.15	43.37	44.81	44.09	49.68	49.02
**100**	26.74	42.40	52.83	52.83	48.19	53.05
**150**	27.03	43.96	47.63	46.92	44.53	47.33
**200**	25.09	40.85	48.86	57.26	41.85	47.23
**250**	26.43	38.76	48.75	41.93	40.21	47.21
**300**	28.92	40.15	49.58	40.41	39.31	46.77
**Mean**	26.74	42.37	46.35	43.91	42.86	45.58

Table S2. Results of neural prosthesis control by electrically induced BI-R signals.

Trial	Stimulus intensity (μV)	Response (μA)	Threshold (μV)	Target	Working
1	150	3310	2148	O	O
2	150	3320	2148	O	O
3	150	3520	2148	O	O
4	150	3200	2148	O	O
5	150	3120	2148	O	O
6	150	3520	2148	O	O
7	150	3150	2148	O	O
8	150	2820	2148	O	O
9	150	2800	2148	O	O
10	150	3200	2148	O	O
11	130	1200	2148	X	X
12	130	1600	2148	X	X
13	130	1800	2148	X	X
14	130	1200	2148	X	X
15	130	1420	2148	X	X
16	130	1300	2148	X	X
17	130	1250	2148	X	X
18	130	1180	2148	X	X
19	130	1200	2148	X	X
20	130	1150	2148	X	X

Experimental section

Neural prosthetic control through electrically evoked BI-R signals: After hook electrodes were implanted in the sciatic nerve, the efferent RPNI signals induced from the BI-R of the bionic interface were recorded in real time through electrical stimulation. The stimulation parameters were a pulse width of 50 μs and an interpulse delay of 10 μs, and a total of 10 stimulations were performed with an intensity of 150 μA.

At the same time, the recorded signal was smoothed by passing through a bandpass filter with a cutoff frequency of 1 to 3500 Hz and a Savitzky–Golay filter to remove noise and to drive the lower limb prosthetics when the evoked RPNI muscle signal was over the threshold (30σ_noise). The RMS of the noise was calculated for 5 seconds of recording in the absence of any stimulus. In addition, to check whether negative working is possible when the bionic interface is used to obtain input data for neural prosthetic control, the stimulation intensity was lowered to 130 μA so that the RPNI signal below the threshold was induced, and a total of 10 simulations were performed. Differential bipolar recording was performed using the two channels located on the BI-R as the working and reference electrodes and the stainless-steel wire attached to the rabbit ear as the ground electrode. Signals were recorded in real time through an electrophysiology amplifier (RHD 2216; Intan Technology) **(Figure S9)**.

Result & Discussion

Neural prosthetic control through electrically evoked BI-R signals: In both positive and negative tests, it was confirmed that the control of the neural prosthesis was possible with 100% accuracy through the electrically induced BI-R signal **(Table S2)**. It was confirmed that the neural prosthesis worked with all BI-R signals induced above the threshold value, and the neural prosthesis did not operate with BI-R signals induced below the threshold value. These results demonstrate the potential for neural prosthetic control through bionic interfaces. Further experiments on controlling neural prostheses using both BI-R and BI-N signals as well as naturally evoked somatic signals are needed to further demonstrate the potential of the bionic interface.
